# Implementing the First Outpatient Parenteral Antimicrobial Therapy (OPAT) Program to Utilize Disposable Elastomeric Pumps in the Gulf Region: Results From a Tertiary Teaching Hospital in the Kingdom of Saudi Arabia

**DOI:** 10.7759/cureus.20179

**Published:** 2021-12-05

**Authors:** Ahmed Zikri, Hassan Al-Faraj, Nabil Kamas, Jumaan AlZahrani, Hisham BuKhamseen, Wasan Alshahoub, Arlene Beltran, Dalia Fatih, Zainab AlMusa

**Affiliations:** 1 Department of Pharmacy, King Fahad Specialist Hospital, Dammam, SAU; 2 Department of Internal Medicine, King Fahad Specialist Hospital, Dammam, SAU; 3 Department of Home Medicine, King Fahad Specialist Hospital, Dammam, SAU; 4 Department of Internal Medicine/Infectious Diseases, King Fahad Specialist Hospital, Dammam, SAU

**Keywords:** home health care, antibiotics, elastomeric pumps, outpatients intravenous antimicrobial therapy (opat), infections

## Abstract

Objectives

To describe the implementation process, safety, and efficacy outcomes, as well as cost-effectiveness, of the first outpatient parenteral antimicrobial therapy (OPAT) program to utilize disposable elastomeric pumps in the Kingdom of Saudi Arabia and the entire Gulf region.

Methods

This OPAT program was initiated in May 2018 and was administered through a multidisciplinary team that included the home medicine department, pharmacy department, nursing department, and the infectious diseases service. The device used was the Intermate® (Baxter, Deerfield, Illinois) elastomeric pump. After consultation with an infectious diseases physician, eligible patients were discharged home to complete the remainder of their antimicrobial treatment, which was self-administered via the elastomeric devices.

Results

From May 2018 to December 2019, 47 patients received 55 courses of OPAT via the new program. A total of 2,869 pumps were used during that period to provide 927 days of antimicrobial therapy in the home setting. Most patients completed the program successfully with no reported significant OPAT-related complications such as catheter-related infections. Four patients were re-admitted for relapse of infections and one patient was re-admitted for colistin-induced nephrotoxicity. No mortality was reported for any patient during OPAT treatment and 30 days after program completion.

Conclusions

The implementation of this novel OPAT program was safe, effective, and offered significant cost-savings to our institution. The entire process was very dynamic and was centered around proper patient selection and education as well as excellent communication between patients and the entire multidisciplinary team involved in the program. We hope that these results will encourage other institutions in the region to implement similar OPAT programs to alleviate the existing bed crisis due to the ongoing COVID-19 pandemic.

## Introduction

Outpatient parenteral antimicrobial therapy (OPAT) is defined as the administration of parenteral antimicrobial therapy in at least two doses on different days without intervening hospitalization [1]. Some infectious diseases such as endocarditis and osteomyelitis may require treatment with prolonged courses of intravenous antimicrobial therapy. Moreover, the emergence of antimicrobial resistance may require the use of intravenous agents for the entire duration of therapy if the causative organism is not susceptible to any of the available oral antimicrobial options.

In 1974, Rucker and colleagues described the first program to successfully use OPAT to treat chronic bronchopulmonary infections in children with cystic fibrosis [2]. Since then, multiple studies have demonstrated that OPAT programs can decrease the rates of healthcare-associated infections while improving patient satisfaction and overall hospital experience [3-7].

OPAT has become a well-established practice worldwide with significant program implementation growth over the past couple of decades [3,5,6,8-11]. The safety and efficacy of OPAT programs have been well documented in numerous studies, with success rates exceeding 90% [3,5]. Proper patient selection for the program was linked to improved safety, even while treating complicated infections such as endocarditis [6,9,12].

The Infectious Diseases Society of America (IDSA) published its first OPAT guidelines in 2004, which were revised and updated in 2018. The guidelines provide recommendations regarding appropriate patient evaluation and selection, antimicrobial utilization and stewardship, and clinical monitoring [1].

OPAT programs can offer significant benefits for both healthcare systems and patients. Healthcare systems can benefit from preventing healthcare-associated infections, shorter or avoided hospitalizations, and significant cost savings. For patients, OPAT programs allow them to return to work or school faster, care for children or dependents, and resume daily living activities with minimal interruptions in their lives [1].

In Saudi Arabia, the concept of OPAT has not been widely accepted, despite the worldwide implementation of such programs. However, there has been more interest in recent years to utilize OPAT programs to shift care to the outpatient setting to improve quality of life and alleviate the increasing need for acute care beds to care for a growing population. The need to establish safe, efficacious, convenient, and cost-effective OPAT programs is increasingly recognized in the Kingdom. In this pilot study, we report the first use of elastomeric infusion devices to self-administer OPAT in Saudi Arabia and, most probably, in the entire Gulf region. We describe the implementation process, safety, and efficacy outcomes, as well as cost-effectiveness, of the program.

## Materials and methods

Program initiation and implementation

King Fahad Specialist Hospital is a tertiary referral center in the eastern province of Saudi Arabia with a capacity of 360 beds. When the decision to launch the program in the hospital was made, a failure mode and effect analysis (FMEA) was conducted. During the analysis, it was decided to use a disposable elastomeric device instead of using programmable digital pumps. The pump selected for the project was the Intermate® elastomeric pump (Baxter, Deerfield, Illinois). The final recommendations were sent to hospital leadership, and the project was approved. The home medicine department (HMD) was commissioned to run the program, with support from the pharmacy department, nursing administration, and the infectious diseases consult service. An OPAT policy was drafted by HMD and was reviewed by all involved departments before it was approved by hospital administration and became effective in May 2018.

OPAT procedure

Eligibility criteria include infectious disease diagnosis that requires intravenous antimicrobial therapy in a patient who is otherwise fit for discharge. The patient should be living in a stable home environment and able to understand the implication of the program, and has easy access to medical care when needed. The patient or family member should be willing to learn how to administer the antimicrobial drug. Other requirements are appropriate intravenous access and the use of a stable drug in the OPAT pump. When a primary physician deems a patient eligible for OPAT, he/she discusses the program with the patient and caregivers and gets their approval to start the process. The physician then consults the infectious diseases service to create an OPAT plan. Once a treatment plan is agreed upon, the primary physician consults HMD to provide more education and to conduct a thorough assessment of the patient, caregiver, and home environment to confirm eligibility. A consultation is placed with the vascular access team to insert a peripherally inserted central catheter (PICC) when indicated. A PICC line is inserted for treatment courses longer than one week in duration. A peripheral cannula is inserted for a treatment duration of less than one week.

Once vascular access is inserted, the pharmacy department is contacted to prepare two elastomeric pumps containing the target antimicrobial agent. A nurse administers the first pump to educate the patient and the caregiver on the proper use of the elastomeric device and proper vascular access maintenance techniques. The caregiver administers the second dose to confirm their full understanding of the procedure and demonstrate their ability to use the elastomeric device properly and maintain the patency and sterility of vascular access.

Stability data for antimicrobial therapy in the Intermate® elastomeric pump are obtained through Stabforum® (Baxter, Deerfield, Illinois), a proprietary database owned by Baxter. The database includes stability studies conducted for various antimicrobial agents in the elastomeric device with access to valuable information regarding proper diluent (dextrose 5% in water [D5W] vs. normal saline), concentration range, storage condition (room temperature vs. refrigeration), and duration of stability under these conditions. Most antimicrobial agents are kept in the refrigerator for a longer stability duration. Patients are instructed to take the pumps out of the fridge for 10-15 minutes to allow the solution to reach room temperature before use.

The patient is then discharged home with elastomeric pumps containing the antimicrobial agent and other supplies such as alcohol swaps and dressings for vascular access. HMD schedules a visit to the patient's home a few days after discharge and once weekly after that. The patient is also scheduled to follow up with their primary physician or with an infectious disease physician in the clinic.

Patients are instructed to call the HMD during working hours from Sunday to Thursday for all OPAT-related questions and concerns. If a patient encounters OPAT-related issues after hours or on the weekend, he/she is instructed to visit the emergency department. All patients carry a card indicating that they are receiving OPAT with the name and contact number of the primary physician.

Data collection and analysis

All patients' information and OPAT data were collected from the HMD records, pharmacy records, and the patients' medical records in the hospital's health information system (HIS). Data included patient demographics, comorbidities, referring service, OPAT indication and duration, causative organism, the antimicrobial agent used, type of vascular access, ER visits for OPAT-related issues, safety and efficacy data, and mortality during OPAT administration and 30 days after program completion. All patients who received at least two doses of an antimicrobial agent via an elastomeric infusion pump at home were included in the analysis. King Fahad Specialist Hospital-Dammam Institutional Review Board (IRB) approved the publication of this manuscript (IRB number: IRB-Pub-20-021).

## Results

Patients’ demographics

Since its implementation in May 2018 until December 31st, 2019, 47 patients received OPAT via the new program, with a total of 55 courses of OPAT administered using the Baxter Intermate® elastomeric device. A total of 42 patients (96%) received only one OPAT course, three patients received two courses, one patient received three courses, and one patient received four different courses of OPAT during that period. Thirty patients (64%) were males, while 17 patients (36%) were females. The mean age was 53 years (range 17-77 years).

In regards to comorbidities of enrolled patients, 26 patients (55%) had diabetes, 18 patients (38%) had hypertension, nine patients (9%) had cancer, eight patients (17%) had coronary artery disease, eight patients (17%) underwent a renal transplant, seven patients (15%) had chronic kidney disease, seven patients (15%) had peripheral vascular disease, and only one patient (<2%) underwent a liver transplant surgery.

A total of 45 patients (96%) had no allergies to any antimicrobial agents, while one patient was allergic to penicillin, and another patient was allergic to fluoroquinolones. Regarding vascular access, 32 courses (58%) were administered via a PICC line, 21 courses (28%) were received via peripheral cannula, and two patients received their OPAT courses via a port-a-cath.

Referring services and OPAT initiation sites

Infectious diseases services were consulted on 48 OPAT courses (87%), while seven courses (13%) were started without consultation from an infectious disease physician. The most common referring specialty was the medicine department (24%), followed by vascular surgery (22%), renal transplant service (15%), pulmonology (11%), urology (6%), general surgery department (6%), neurosurgery (4%), and only one referral (<2%) from orthopedic surgery, thoracic surgery, ENT department, gynecology, hematology, hepatobiliary, liver transplant, and infectious diseases service. A total of 37 OPAT episodes (67%) were initiated in the inpatient setting, and 17 episodes (31%) were started in an outpatient setting, with only one episode started in the emergency department. Table 1 summarizes patient demographics, comorbidities, and other OPAT-related information.

**Table 1:** Demographics and general program data. OPAT, outpatient parenteral antimicrobial therapy; PICC, peripherally inserted central catheter.

Total number of patients	47
Males	30 (64%)
Females	17 (36%)
Mean age (range)	53 (17-77)
Comorbidities	
Diabetes	26 (55%)
Hypertension	18 (38%)
Cancer	9 (19%)
Coronary artery disease	8 (17%)
Renal transplant	8 (17%)
Chronic kidney disease	7 (15%)
Peripheral vascular disease	7 (15%)
Liver transplant	1 (2%)
Antibiotic allergies	
None	45 (96%)
Allergies	2 (4%)
- Penicillin	1
- Fluoroquinolones	1
OPAT episodes	55
Patients with one episode	42 (76%)
Patients with two episodes	3 (5%)
Patients with three episodes	1 (<2%)
Patients with four episodes	1 (<2%)
Referring specialty	
Medicine department	13 (24%)
Vascular surgery	12 (22%)
Renal transplant	8 (15%)
Pulmonology	6 (11%)
Urology	3 (6%)
General surgery	3 (6%)
Neurosurgery	2 (4%)
Orthopedic surgery	1 (< 2%)
Thoracic surgery	1 (< 2%)
ENT	1 (< 2%)
Gynecology	1 (<2%)
Hematology	1 (<2%)
Hepato-biliary surgery	1 (<2%)
Liver transplant	1 (<2%)
Infectious diseases	1 (<2%)
Vascular access	
PICC line	32 (58%)
Peripheral line	21 (38%)
Port-a-cath	2 (4%)
Infectious disease consultation	
Yes	48 (87%)
No	7 (13%)
OPAT initiation site	
Inpatient	37 (67%)
Outpatient	17 (31%)
ER	1 (<2%)

Types of infections treated with OPAT

Patients received OPAT courses to treat a variety of bacterial infections. Urinary tract infections were the most commonly treated infection (27%), followed by diabetic foot infections (22%), respiratory tract infections (16%), intra-abdominal infections (9%), and cardiovascular infections (9%). Four OPAT episodes (7%) were used to treat osteomyelitis, three courses (6%) were started for skin and soft tissue infections, and two patients (4%) received OPAT for central nervous system (CNS) infections. Table 2 summarizes sources of infections and their corresponding number and percentage during the study period.

**Table 2:** Infections treated using OPAT program. OPAT, outpatient parenteral antimicrobial therapy; MSSA, methicillin-sensitive *Staphylococcus aureus*; CLABSI, central line-associated bloodstream infection.

Type of infection	Number of patients
Urinary tract infections	15 (27%)
Diabetic foot infections	12 (22%)
- 66% without bone involvement	8
- 34% bone involvement	4
Respiratory tract infections	9 (16%)
- 44% with pneumonia	4
- 44% with bronchiectasis exacerbation	4
- 12% with empyema	1
Intra-abdominal infections	5 (9%)
Cardiovascular infections	5 (9%)
- 60% with MSSA bacteremia	3
- 20% with bacteremia from an unknown source	1
- 20% with CLABSI	1
Osteomyelitis	4 (7%)
Skin and soft tissue infections	3 (6%)
- 66% with cellulitis	2
- 34% with perianal abscess	1
CNS infections	2 (4%)
- 50% with brain abscess	1
- 50% with mastoiditis with subdural collection	1

Causative pathogens for infections

Infections were mostly mono-microbial (75% of all cases), with poly-microbial infections encountered in 18% of all cases, while four episodes (7%) were culture-negative. Gram-negative anaerobes comprised 75% of all causative pathogens, with *Pseudomonas aeruginosa* being the most common organism isolated, followed by *Escherichia coli*, then *Klebsiella pneumoniae*. A total of 14% of *Pseudomonas aeruginosa* strains were multidrug-resistant organisms (MDROs). Rates of extended-spectrum beta-lactamase (ESBL) productions in *E. coli* and *Klebsiella pneumoniae* were 59% and 67%, respectively. Gram-positive aerobes constituted 22% of all pathogens, with *Staphylococcus aureus* comprising the majority of pathogens. A total of 63% of isolated *Staphylococcus aureus* strains were methicillin-sensitive, while the remainder 34% of strains were methicillin-resistant. Table 3 provides a list of all isolated pathogens for all OPAT-treated patients.

**Table 3:** Causative organisms. MDRO, multidrug-resistant organism; ESBL, extended-spectrum beta-lactamase; MSSA, methicillin-sensitive *Staphylococcus aureus*; MRSA, methicillin-resistant *Staphylococcus aureus.*

No. of episodes	55
Mono-bacterial	41 (75%)
Poly-bacterial	10 (18%)
Culture-negative	4 (7%)
No. of organisms	63
Gram negative aerobes	47 (75%)
*Pseudomonas aeruginosa* (45%)	21
- of which 14% MDRO strains	3
*Escherichia coli *(36%)	17
- of which 59% ESBL +ve	10
*Klebsiella pneumoniae* (13%)	6
- of which 67% ESBL +ve	4
*Serratia marcescens* (2%)	1
*Providencia rettgeri* (2%)	1
Salmonella species (non-typhi) (2%)	1
Gram positive aerobes	14 (22%)
*Staphylococcus aureus* (57%)	8
- of which 63% MSSA	5
- of which 34% MRSA	3
*Staphylococcus lugdunensis* (7%)	1
*Streptococcus pyogenes* (7%)	1
*Streptococcus anginosus* (7%)	1
*Enterococcus faecalis* (14%)	2
Actinomyces species (7%)	1
Anaerobic bacteria	2 (3%)
Bacteroides species (50%)	1
Lactobacillus species (50%)	1

Antimicrobial agents used during the study period

Ten different antimicrobial agents were used for OPAT. Most commonly used agent was piperacillin/tazobactam (26%), followed by imipenem (20%), ceftriaxone (16%), meropenem (15%), vancomycin (7%), and then cefazolin and cefepime (5% each). Clindamycin, colistin, and aztreonam were only used once during the study period. A total of 2,869 disposable elastomeric pumps were used to provide 927 days of antimicrobial therapy from program initiation in May 2018 until the end of December 2019. Table 4 provides data on all antimicrobial agents used, the number of days of therapy (DOTs) for each agent, and the total number of elastomeric pumps used to administer each drug throughout the 19-month study period.

**Table 4:** Antimicrobial agents used. DOTs, days of therapy.

Drug	Episodes (%)	DOTs (%)	No. of pumps (%)
Total	55	927	2,869
Piperacillin/tazobactam	14 (26%)	257 (28%)	1,028 (36%)
Imipenem	11 (20%)	162 (18%)	648 (23%)
Ceftriaxone	9 (16%)	159 (17%)	220 (8%)
Meropenem	8 (15%)	123 (13%)	369 (13%)
Vancomycin	4 (7%)	89 (10%)	194 (7%)
Cefazolin	3 (5%)	62 (7%)	186 (6%)
Cefepime	3 (5%)	25 (3%)	75 (3%)
Clindamycin	1 (2%)	37 (4%)	111 (4%)
Colistin	1 (2%)	7 (<1%)	14 (<1%)
Aztreonam	1 (2%)	6 (<1%)	24 (<1%)

Safety data (emergency room visits, clinical failure, and hospital re-admissions)

During the study period, there were 42 emergency room visits for OPAT-related issues. Eighteen visits (43%) were for re-cannulation and 11 visits (27%) were for vascular access patency. Five visits (12%) were for vascular access dressing change, and one patient visited the emergency department for vascular access removal. Six visits were for relapse symptoms, and four patients were re-admitted for inpatient treatment. One patient visited the emergency department for acute kidney injury (AKI), secondary to colistin therapy. The drug was discontinued, and the patient was re-admitted. AKI was mild and was reversed upon discontinuation of colistin. Out of the 47 patients who received OPAT during that period, four patients (8.5%) were re-admitted for relapse of infection, and one patient (2%) was admitted for colistin-induced nephrotoxicity. No mortality was reported for any of the 47 OPAT patients during treatment and 30 days after OPAT discontinuation. Table 5 lists all emergency department visits as well as OPAT-related re-admissions.

**Table 5:** Emergency room visits and OPAT-related re-admissions. OPAT, outpatient parenteral antimicrobial therapy.

OPAT-related ER visits	42 visits
Re-cannulation	18 (43%)
Vascular access complication	11 (27%)
Relapse symptoms	6 (14%)
Vascular access dressing	5 (12%)
Acute kidney injury secondary to colistin	1 (2%)
Vascular access removal	1 (2%)
OPAT-related re-admission	5 patients
Relapse	4 patients (80%)
Acute kidney injury secondary to colistin	1 patient (20%)

Cost benefits of the new OPAT program

The new OPAT program provided significant direct cost benefits to our institution. From its implementation in May 2018 until the end of December 2019, 47 patients received 55 OPAT courses, with 927 DOTs. The projected cost of all 927 days of admission to receive treatment in the inpatient setting, in the absence of an OPAT program, would have been Saudi riyal (SR) 3,708,000. The total cost of elastomeric pumps used during that time was SR 393,053. The total cost for all HMD visits to patients' homes during working hours and patients' visits to the emergency department after working hours and on weekends was SR 555,053. The direct cost saving of the program during the 19-month trial period was SR 3,152,947. Table 6 summarizes the cost-benefit analysis of the new program.

**Table 6:** Cost-benefits analysis of the new OPAT program. SR, Saudi riyal; HMD, home medicine department.

No. of patients	47
No. of episodes	55
Days of therapy (DOTs)	927
Cost per day	SR 4,000
Total cost for 927 DOTs	SR 3,708,000
Number of pumps	2,869
Cost per pump	SR 137
Total cost of pumps	SR 393,053
Number of home visits by HMD	174
Cost per HMD visit	SR 750
Total cost of all HMD visits	SR 130,500
Number of ER visits	42
Cost per ER visit	SR 750
Total cost of all ER visits	SR 31,500
Cost of pumps and HMD and ER visits	SR 555,053
Direct cost savings	SR 3,152,947
Direct return on investment (ROI)	668%

## Discussion

OPAT programs have become the standard of care in many countries throughout the world. There are multiple studies in the literature supporting the safety and efficacy of OPAT programs. In countries where home OPAT programs are widely used, mainly in Europe and North America, an extensive healthcare infrastructure ensures such programs' success.

In Middle Eastern countries and other parts of the world, the lack of proper infrastructure makes it more challenging to implement these programs. Lack of essential resources such as trained nursing staff and programmable digital infusion pumps makes it difficult for healthcare providers to discharge patients earlier and allow them to complete their intravenous antimicrobial therapy in their homes. This often leads to extended hospitalization, prolonged length of stay, and increased risk of healthcare-associated complications, including healthcare-acquired infections. An additional challenge to implementation is the fear of sending patients home with vascular access due to concerns for the development of catheter-related infections.

There are only a handful of studies in the literature describing the use of OPAT programs in the entire Gulf region. In 2011, Baharoon and colleagues described the successful implementation of a home-based OPAT program in Riyadh, where 152 patients were successfully treated at home from May 2005 until December 2007. The program did involve daily visits from a home health nurse to administer each dose of antibiotics via programmable infusion pumps. A total of 68 patients received three visits per day, while 60 patients received two visits per day, and only 24 patients received home visits once daily [13].

In another study published in 2013, Ansari et al. reported their experience with a clinic-based OPAT program that ran from February 2012 until January 2013 in the Kingdom of Bahrain. During that time, 101 patients, 92 adults, and nine children were treated in an outpatient clinic after a referral from inpatient and outpatient services. A total of 76% of patients received once-daily ceftriaxone. Other antibiotics requiring more frequent administration, such as meropenem, piperacillin/tazobactam, and vancomycin, were used much less frequently due to the difficulty of administering multiple doses of antibiotics in a clinic setting [14].

A more effective and convenient approach is a home-based OPAT program, where antimicrobials are self-administered by patients using disposable elastomeric infusion devices. This modality does not require nursing staff to visit the patient's home to administer every dose or the use of programmable digital pumps. Once a patient and his/her caregiver receive proper training on administering their antimicrobial agent using the elastomeric device, they can be discharged home to self-administer the remainder of their antimicrobial treatment course.

To our knowledge, our center is the first hospital to implement an OPAT program using disposable elastomeric devices in the Kingdom of Saudi Arabia and potentially in the entire Gulf region. The program's implementation was successful with excellent safety and efficacy data and significant cost-saving benefits. Since the implementation of our program, other institutions in the region started considering establishing similar OPAT programs.

Patient safety was the most crucial consideration once the decision was made to launch an OPAT program. The data show that the program was very safe with no significant reported complications such as catheter-related bloodstream infections. No mortality was reported during the administration of OPAT or for 28 days after program completion. The majority of ER visits were for minor issues such as re-cannulation, vascular catheter occlusion, or catheter dressing change. Only four patients (8.5%) were re-admitted for relapse of infection and were successfully treated in the inpatient setting. One patient was re-admitted for colistin-induced nephrotoxicity, which was mild and quickly reversible upon discontinuation of the medication.

Our OPAT program was also very effective. The majority of patients completed their treatment course with no issues. Most patients were very satisfied with the program and many of them expressed appreciation for the ability to complete their antimicrobial course in the comfort of their homes. One patient, who is bedridden and is on mechanical ventilation, was extremely grateful that he could go home to finish his treatment course instead of staying in the hospital for the entire duration of treatment.

One of the most significant benefits of the program is its cost-saving potential. During the 19 months of this pilot study, our OPAT program delivered 927 DOTs to 47 patients. The cost of inpatient hospitalization for these 927 DOTs is estimated at SR 3,314,947, based on a daily cost of SR 4,000 per day. During the same time, HMD paid 174 follow-up visits to patients’ homes. Some patients who only received a few days of OPAT did not need any home visits. Others who received a longer course were visited up to 10 times during their home treatment. This represents an average of approximately three HMD visits per OPAT course. It also translates to one HMD visit for every 5.3 days of avoided hospitalization. The cost of each HMD visit that does not involve the administration of medications is SR 750. The total cost of all 174 visits was SR 130,500. There were also 42 emergency department visits, most of which were for re-cannulation or line patency issues. The average cost per those visits was SR 750 per visit, with a total cost of 31,500 for all 42 visits. The total cost of the pumps, in addition to the cost of HMD visits to patients’ homes and patients’ visits to the emergency department, was SR 555,053. When considering the cost of avoided hospitalization for all 927 DOTs, this translates to a cost saving of SR 3,152,947. The direct return on investment (ROI) for this program was 668%.

In addition to direct cost benefits, there were also significant indirect cost benefits realized from program implementation. The program's most significant benefit is alleviating the existing bed crisis and improving bed utilization to provide care to as many patients as possible. Another benefit is reducing the incidence of healthcare-associated complications, including healthcare-acquired infections. A third indirect benefit is improving patients' quality of life through expedited discharge and providing them the opportunity to complete their intravenous antimicrobial treatment from the comfort of their homes. Unfortunately, proper metrics were not available to adequately calculate the indirect cost of these benefits.

The implementation was met with some challenges. First, the decision to use elastomeric pumps meant that an additional budget to purchase these devices had to be allocated. The hospital administration did not initially agree to provide funding for purchasing the elastomeric pumps. However, after presenting data on the potential cost savings by decreasing the number of admissions, reducing the length of stay, and minimizing healthcare-associated complications, the hospital administration agreed to finance the program.

Another challenge was identifying clear roles and responsibilities for all involved departments and services. Our OPAT program is a multidisciplinary effort championed by the HMD and fully supported by the pharmacy department, nursing administration, and the infectious diseases service. It was vital for our program's success to create an OPAT policy that clearly defined the program's scope and identified the roles and responsibilities of every concerned service. This was challenging initially, but through continuous communication and constant adjustments, we were able to streamline initiating OPAT and provide adequate follow-up until the treatment course was successfully completed.

A third challenge was related to the safety of the program itself. Many clinicians and patients alike were concerned that administering intravenous antimicrobial therapy in the home setting by the patient and their caregivers may increase the risk of catheter-related infections. This was challenging initially as many clinicians who were eager to send their patients home were reluctant to use the program due to the safety concerns mentioned above. This issue was resolved through education. We explained the process to physicians and stressed that patients and caregivers are provided with a comprehensive education on the proper use of the pumps and must demonstrate full competency before being discharged home.

The final challenge was regarding stability data of antimicrobials in the elastomeric pumps. Most antimicrobial agents have longer stability data, up to 28 days, when kept refrigerated. A one-week course was prepared for those agents, and caregivers picked up the pumps once a week. For some agents with poor stability data, such as meropenem and imipenem, which are only stable for 24 hours when refrigerated, the pumps had to be prepared daily, and a caregiver had to pick them up every day. Despite the daily visits to collect the pumps, most patients and their caregivers still preferred going home on the new OPAT program instead of staying at the hospital for the entire treatment duration.

On a final note, a crucial decision that greatly enhanced the program's safety and efficacy was requiring the infectious diseases service's involvement. IDSA OPAT guidelines state that all patients should have an infectious diseases expert review before initiating OPAT as a strong recommendation [1]. The vital role of infectious diseases physicians in the antimicrobial selection, antimicrobial stewardship, improving patients' outcomes, and decreasing cost is well documented in the literature. In OPAT programs that included an infectious diseases physician consultation, switching to oral antibiotics and stopping antibiotics altogether occurred 25% and 10% of the time, respectively [15,16]. This crucial involvement can improve clinical outcomes, decrease antimicrobial resistance, and reduce the cost of treatment. Other studies demonstrated that the involvement of an infectious diseases physician in these programs has reduced re-admissions and emergency room visits [17-19].

## Conclusions

The implementation of the first OPAT program to utilize disposable elastomeric devices in the Kingdom of Saudi Arabia was safe and effective and offered significant cost savings to our institution. Program implementation was a very dynamic process, which was centered around providing extensive education to both patients and healthcare providers and proper communication between patients and the entire multidisciplinary team involved in the care process. We sincerely hope that our study results would encourage other healthcare institutions in the region to consider implementing similar OPAT programs that are tailored to their available resources and their patient-specific needs.
